# Twelve Weeks of Strengthening Exercise for Patients with Rheumatoid Arthritis: A Prospective Intervention Study

**DOI:** 10.3390/jcm9092792

**Published:** 2020-08-29

**Authors:** Bomi Sul, Kyoung Bo Lee, Young Bin Joo, Bo Young Hong, Joon-Sung Kim, Ki-Jo Kim, Kyung-Su Park, Yune-Jung Park, Seong Hoon Lim

**Affiliations:** 1 Department of Rehabilitation Medicine, St. Vincent’s Hospital, College of Medicine, The Catholic University of Korea, Seoul 06591, Korea; snowspringee@naver.com (B.S.); kblee0732@naver.com (K.B.L.); byhong@catholic.ac.kr (B.Y.H.); svpmr@chol.com (J.-S.K.); 2 Division of Rheumatology, Department of Internal Medicine, St. Vincent’s Hospital, College of Medicine, The Catholic University of Korea, Seoul 06591, Korea; addmedic@hanmail.net (Y.B.J.); md21c@catholic.ac.kr (K.-J.K.); pkyungsu@catholic.ac.kr (K.-S.P.)

**Keywords:** rheumatoid arthritis, RA, strengthening exercise, arthritis, exercise, mental heath

## Abstract

Rheumatoid arthritis (RA) patients may benefit from exercise for several reasons. However, whole-limb strengthening exercises for such patients remain poorly studied. We hypothesized that systemic strength training that includes the upper and lower extremities would improve strength per se and enhance the quality of life. Here, we investigated the effects of 12 weeks of upper- and lower-limb strengthening exercise on the strength and quality of life of RA patients using the International Classification of Functioning, Disability, and Health model. This was a prospective, interventional controlled trial. Forty female RA patients were recruited and assigned to two groups not based on willingness to exercise, with 20 patients in the exercise group and 20 in the control group. All patients in the exercise group received once-weekly training sessions of 60 min over 12 weeks. All participants were assessed before and after the 12-week intervention period. We measured the hand grip strength and isometric quadriceps contraction, the cross-sectional area of the rectus femoris (CSA-RF) (via ultrasonography), and performed the 30 s sit-to-stand test and the 6 min walk test (6MWT). We derived the Borg scale score after the 6MWT and assessed the extent of social participation and quality of life using a Korean version of the 36-Item Short Form Health Survey (SF-36). A total of 35 subjects completed the experiment (18 in the exercise group, 17 in the control group). After the 12-week intervention period, the lower-limb strength and the CSA-RF were significantly increased in the exercise group. The activity level did not change significantly in either group. The exercise group exhibited significant improvements in the SF-36 mental health domain scores. Thus, strengthening exercise is useful for patients with RA.

## 1. Introduction

Rheumatoid arthritis (RA) is a chronic, autoimmune, inflammatory systemic disease that principally affects the joints. RA is usually treated pharmacologically; exercise is an adjunct option [1]. Aerobic and resistance exercises increase cardiorespiratory fitness and reduce the risk of cardiovascular disease and disease activity and severity in RA patients [2]. Hand strengthening and stretching exercises were found to be clinically beneficial and cost-effective for patients with RA in [3].

Regarding investigations on strengthening exercises, one supervised long-term group exercise program consisted of a bicycle and exercise circuit. It resulted in improved functional ability, based on the MACTAR questionnaire, in patients with RA [4]. The home exercise program was also beneficial for physical activity, and the effectiveness of the exercise program continued up to 12 months after interventions [5,6]. Most studies on strengthening exercise include a bicycle program. The bicycle is one of the tools used for aerobic and strengthening training. However, cultural or environmental differences might be a barrier to the use of bicycles. Moreover, physical activity per se afforded many benefits [7]. Moreover, the effects of whole-limb strengthening exercise remain poorly studied. Thus, we designed a home strengthening exercise program consisting of just manual and elastic bands in order to ensure accessibility.

We hypothesized that systemic strength training that includes the upper and lower extremities would improve strength per se and enhance the quality of life. We evaluated the effects of 12 weeks of upper- and lower-limb strengthening exercise with elastic bands on the strength and quality of life of RA patients using the International Classification of Functioning, Disability, and Health (ICF) model.

## 2. Material and Methods

### 2.1. Participants

**Figure 1:**
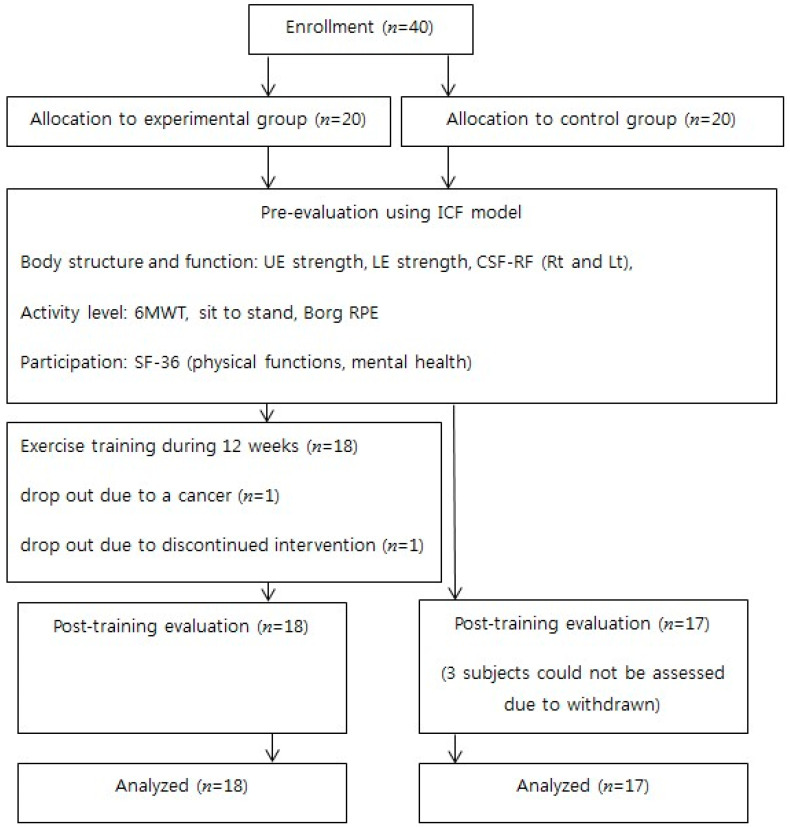
Subject flowchart. *n*, Number; ICF, International Classification of Functioning, Disability, and Health; UE, Upper Extremity; LE, Lower Extremity; CSA-RF, The cross-sectional area of the rectus femoris; 6mwt, 6-min walk test; RPE, Rating of Perceived Exertion.

This was a prospective, interventional controlled trial. Female RA patients were consecutively recruited from the rheumatology department of St. Vincent’s Hospital. The inclusion criteria were age > 18 years, a sedentary lifestyle (no participation in structured exercise over the preceding 3 months), and stable disease (no changes in disease-modifying anti-rheumatic drugs or steroids in the last 3 months). Patients with an inability to bear weight on their lower extremities, a history of hip or knee replacement surgery, a recent or ongoing disease flare, an unstable heart condition (ischemic heart disease during the last month, heart rate > 120/min at rest, systolic blood pressure > 180 mmHg, or diastolic blood pressure > 120 mmHg), or serious comorbidities (e.g., malignancy) were excluded. One rheumatologist performed a screening of disease activity based on clinical and laboratory data. Stable disease defined as a change in Disease Activity Score (DAS28) was ≤3.2 (low current disease activity), and the difference in DAS28 scores between the baseline and the last measurement was ≤1.2 [8]. The patient flowchart is shown in Figure 1. Forty patients were recruited and assigned to two groups not based on their willingness to participate in exercise, with 20 in the exercise group and 20 in the control. Two subjects in the exercise group dropped out (after a diagnosis of breast cancer and for a personal reason). Three control subjects dropped out within 2 weeks for personal reasons.

The study was conducted in accordance with the Declaration of Helsinki, and the protocol was approved by the Ethics Committee of the Catholic University of Korea (VC18FESI0049). The procedures were fully explained to all subjects, and written informed consent was obtained.

### 2.2. Exercise Program

All patients in the exercise group received once-weekly training sessions over 12 weeks. They were provided with handouts and video clips containing instructions and descriptions of the exercises learnt, and were encouraged to repeat the exercises at home at least twice weekly. Attendance was recorded. Instructions were provided, and the patients were supervised during all the sessions by a professional exercise physiologist. Each exercise session lasted for 60 min, commencing with 15 min of warm-up stretching, followed by 45 min of resistive exercise using elastic bands of different strengths (TheraBand, Akron, OH, USA; yellow, red, and green in color) (Table 1). The resistance exercises targeted the major muscle groups based on the guidelines of the American College of Sports Medicine (ACSM) for older adults [9]. Exercise intensity was progressively increased by increasing the resistance of the elastic band (based on the TheraBand force–elongation table) as reflected by a color progression from yellow to red and then to green [10]. The exercises included resistance band squats, bends and rowing, standing alternate chest presses, diagonal swing lifts, triceps extensions, lunges, lateral rowing, and biceps curls. Two sets of 15 repetitions were completed for each exercise, with a rest of 2 min between exercises. During the first four sessions, all subjects wore yellow TheraBands while learning the exercises and adapting to the training. After the four sessions, those who had performed two complete sets of most exercises and were not severely fatigued were given red TheraBands. The others continued exercise with the yellow bands. The participants who tolerated the red TheraBands performed exercises using green bands after another four sessions [10]. In summary, when the patient was tolerable for the strength of one, we prescribed a stronger one; yellow, red, green. The patients returned to the training session with their daily checklist. They were encouraged to perform the task, the daily 60 min exercise, 5 days per week. We used verbal encouragement and daily text messaging via cellular phones.

All the control patients were instructed not to commence an active exercise program that might change their level of physical activity; however, they were allowed to continue pre-existing recreational activities other than resistance or systemic aerobic exercise.

### 2.3. Assessments

All participants were assessed before and after the 12-week intervention period.

#### 2.3.1. Body Structure and Function

We evaluated upper- and lower-extremity muscle strength by measuring (both) hand grip strengths by employing an electronic hand grip meter (model no. KH-100, Kyung In., Korea) with the subjects seated, the shoulders adducted in neutral rotation, and the elbow at 90° flexion. All measurements were performed three times, and the data averaged. We assessed isometric quadriceps contraction using a handheld dynamometer (JTECH MEDICAL Inc., Midvale, UT, USA). All subjects were seated upright in a chair with back support and the knee placed at 90° flexion [11]; they then performed three maximal isometric quadriceps contractions on either side. Averages were recorded. All assessments were conducted by the trained experienced therapist who supervised the exercise sessions.

The cross-sectional area of the rectus femoris (CSA-RF) was ultrasonographically measured using an 8–13 MHz linear array transducer (Accuvix XQ, Samsung Medison, Seoul, Korea) placed perpendicular to the long axis of the thigh (the superior aspect), three-quarters of the distance from the anterior superior iliac spine to the superior aspect of the patellar border [12]. The knee was at rest in full extension. Minimal compression was imparted by coating the transducer with gel, and the smallest possible CSA-RF was measured. The ultrasonographic assessments were performed by one physiatrist blinded to the exercise program.

#### 2.3.2. Activity

The objective outcomes included the results of the 30 s sit-to-stand (STS) test [13], the 6 min walk test (6MWT), and the Borg scale score after the 6MWT. The STS test is widely used to measure lower-limb strength. We required the participants to stand up from and sit down on a 42 cm high armless chair as quickly as possible over 30 s. The shorter time of two trials was recorded. The 6MWT measures the maximum distance walked over 6 min, thus assessing endurance and mobility. The participants were instructed to walk back and forth along a 20 m walkway. Rests were taken when necessary. The evaluators provided standard encouragement every 30 s. All participants performed two trials with as much rest as needed between trials, and the best performance was recorded [14]. Immediately after the 6MWT, the Borg scale score (0–10) was used to rate perceived loading or relative respiratory distress [15]. Assessments were conducted by the same physiologist who evaluated grip strength and quadriceps power.

#### 2.3.3. Social Participation and Quality of Life

The extent of social participation and the quality of life were assessed using a validated Korean version of the 36-Item Short Form Health Survey (SF-36) [16]. This features 1 question on any change in health status and 35 additional questions divided into 8 subscales. We collected the scores of the physical function and mental health domains. The general quality of life was evaluated using the SF-36 norm-based scores [17,18].

### 2.4. Statistical Analyses

Descriptive statistics are presented as frequencies. We explored data normality. Parametric and nonparametric statistics were used to compare group outcomes. We used the paired *t*-test to compare changes within groups. Between-group changes in the outcomes after training were evaluated using a general linear model (analysis of covariance, ANCOVA) after adjusting for the baseline values of the dependent variables (age, CSA-RF (Rt and Lt), and upper-extremity strength). The prediction of sample size had several difficulties. The effect size of strengthening exercise might be difficult to address, as well as exercise itself. We designed the home exercise with face-to-face exercise as a transitional exercise from clinic to home. Thus, we calculated the sample size not based on the previous internet-based population study but on a face-to-face resistance exercise study [6,19]. Based on a previous study on resistance exercise, we calculated a sample size of 20 subjects per group, given an anticipated dropout rate of 15% [19]. All data analyses were performed with the aid of IBM SPSS Statistics, version 21.0 (IBM Corp., Armonk, NY, USA) and a *p*-value < 0.05 was considered statistically significant.

## 3. Results

### 3.1. Baseline Characteristics and Clinical Data

Figure 1 shows the study flow diagram. Training was not associated with any adverse event. The demographic and pre-training test characteristics of all subjects are shown in Table 2. In terms of body structure and functional variables, the groups differed significantly in age, upper-extremity strength (Rt), and CSA-RF (Rt and Lt). However, the 6MWT, STS, Borg, physical function, and mental health measures were similar between the groups.

### 3.2. Post-Training Change

#### 3.2.1. Body Structure and Function

**Figure 2:**
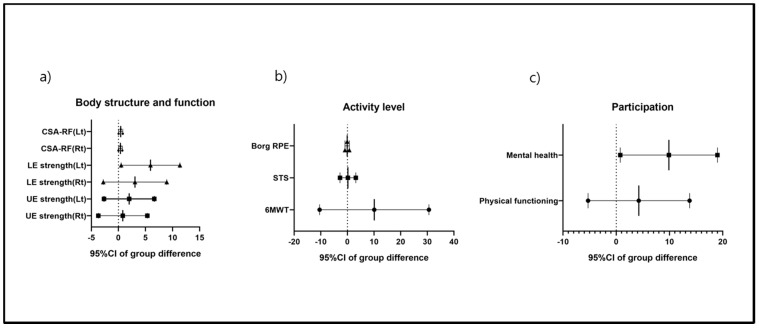
The means with 95%CIs of the between-group differences after the training. (**a**) Body function and structure, (**b**) activity level, and (**c**) participation.

After a 12-week intervention to improve upper-extremity strength, neither the exercise group nor the control group exhibited a significant improvement. Lower-extremity strength improved only in the exercise group. Only the Lt lower-extremity strength differed significantly between the groups. The CSA-RF improved on both sides only in the exercise group. However, although the baseline values differed, the exercise group exhibited significant improvements both within and between the two groups (Table 3, Figure 2a).

#### 3.2.2. Activity Levels

The exercise- and control-group 6MWT and STS scores improved from the baseline values but did not differ significantly between the groups. The Borg scale score did not decrease significantly within or between the groups (Table 3, Figure 2b).

#### 3.2.3. Participation

The exercise group exhibited significantly increased physical function and improved mental health following the 12-week training period; mental health improved only in that group (Table 3, Figure 2c).

## 4. Discussion

Our hypothesis was that systemic strength training, including the upper and lower extremities, would improve strength per se, and enhance the quality of life of patients with RA. We explored the effects of 12-week upper- and lower-limb strengthening exercises on the strength and quality of life in RA patients using the ICF model. A recent Cochrane review showed that exercise improved land-based aerobic capacity and muscle strength, and moderately enhanced aerobic capacity and aerobic muscle strength [20]. We also found that exercise improved muscle strength; in addition, systemic strength training improved the quality of life of RA patients, as revealed by the ICF model. Systemic upper- and lower-limb strength training should be added to the best-practice usual care for RA patients.

In patients with mechanical pain syndrome (e.g., low back pain), the merits of strengthening exercises are well known [21,22]. However, such exercises are not routinely performed by RA patients. Exercise programs for such patients have focused on reducing the cardiovascular risk and improving psychological health [1]. However, recent studies have shown that in RA patients, resistance exercise may improve joint mobility and reduce cartilage breakdown by affecting the expression levels of the interleukin (IL)-1 receptor antagonist and IL-10 [19,20,23]. Taken together with our results, strengthening exercise would increase joint mobility and strength, and improve the quality of life of RA patients. In other words, the strengthening exercise may improve the activity of the disease, physical performance, and the quality of life of RA patients.

For RA patients, atlantoaxial subluxation is an important issue for complication and prescribing exercise programs [24]. The level of anti-citrullinated protein antibody has been known as a predictor of the atlantoaxial subluxation [25]. Thus, the recent decline in the prevalence of the atlantoaxial subluxation has been suggested to result from the use of disease-modifying anti-rheumatic drug therapies [24,25]. The isometric neck flexion exercise is also useful for the stabilization of the atlantoaxial subluxation [26]. Our study aimed to investigate the effects of strengthening exercise. We excluded atlantoaxial subluxation as the reason for an inability to bear weight on subjects’ lower extremities.

The limitations of this study include the lack of randomization, blindness, and small sample size. In the early phase of this study, we quit randomization due to the withdrawal of control subjects. We stopped the randomization and changed the trial. The groups were divided by enrolling order by a rheumatologist by matching the numbers of each group, and he was blinded to all the assessments. In other words, regardless of the patients’ willingness, the process of enrollment was performed. However, this grouping method was not fit for randomization. Thus, we described the study design as a prospective, interventional study. In addition, the intervention of exercise was not fit for a blinded study. Finally, the sample size was small. Prior to our study, the effect size of the whole-limb strengthening exercise did not reach a presumption. Thus, other small effects of strengthening exercise could possibly be neglected by the small sample size. A further population-based study with whole-limb strengthening exercise will be needed to address the remaining questions. The strength of our study is that the exercise program (generalized systematic strength training) is easily performed and increases the quality of life. The program did not improve strength per se or activity. A longer exercise period, and/or the addition of aerobic exercise, may improve strength per se and the activity level.

## 5. Conclusions

The 12 weeks of whole-body strengthening exercise improved lower-limb strength and SF-36-scored mental health. Such exercise is a useful adjuvant treatment for RA patients.

## Figures and Tables

**Table 1:** The exercise program.

**Warm-Up Stretch Exercises**	Shoulder and deltoid stretches	Duration and intensity
	Biceps and wrist flexor stretches	2 repeats, 15 min
	Quadriceps stretch	
	Hamstring and low back stretches	
	Groin stretch	
	Calf stretch	
	Upper back stretch	
	Neck flexor stretch	
	Neck rotator stretch	
**Resistive Band Exercises**	Resistance band squats	15 repeats, 3 sets, 45 min;
	Resistance band bent-over rowing	rest of 2 min between exercises
	Standing alternate chest presses	
	Diagonal woodchops	
	Triceps extension with resistance band	
	Resistance band lunges	
	Lateral rowing with resistance band	
	Biceps curls with resistance band	

**Table 2:** Baseline demographics and clinical characteristics in the enrolled subjects.

Outcomes	EG (*n* = 18) Mean (SD)/Median (IQR)	CG (*n* = 17) Mean (SD)/Median (IQR)	*p*-Value
Height (cm)	158.3 (4.7)		0.853
	158.1 (4.7)	158.4 (4.8)	
Age (years)	50.4 (7.9)		0.015 *
	53.6 (5.2)	47.1 (9.0)	
Weight (kg)	59.8 (9.3)		0.898
	60 (9.2)	59.59 (9.7)	
BMI (kg/m^2^)	24.0 (3.6)		0.879
	24.1 (3.8)	23.9 (3.6)	
Disease duration (years)	5.6 (7.0)		0.987
	5.3 (6.9)	6.0 (7.3)	
**Body function** **and structure**			
UE strength (Rt)	43.4 (12.4)	51.0 (8.6)	0.046 *
UE strength (Lt)	44.4 (8.9)	47.2 (8.9)	−0.366
LE strength (Rt)	55.1 (7.2)	54.4 (6.5)	0.768
LE strength (Lt)	54.3 (6.4)	53.0 (5.6)	0.508
CSA-RF (Rt)	1.0 (0.3)	1.4 (0.8)	0.043 *
CSA-RF (Lt)	0.9 (0.2)	1.4 (0.8)	0.025 *
**Activity level**			
6MWT	508.6 (48.5)	494.7 (49.9)	0.407
STS	15.5 (14.8:19.3)	15.0 (14.0:18.0)	0.499
Borg	2.0 (1.8:3.0)	2.0 (1.5:3.0)	0.884
**Participation**			
Physical function	71.4 (12.5)	72.7 (17.0)	0.803
Mental health	53.9 (9.8)	58.6 (6.8)	0.111

* Significantly different (*p* < 0.05). EG, experimental group; CG, control group; UE, upper extremity; LE, lower extremity; CSA-RF, cross-sectional area of the rectus femoris.

**Table 3:** Results of the paired *t*-tests and ANCOVA used to compare the changes after training within and between groups.

Outcomes	EG (*n* = 18) Mean Change (95%CI)	CG (*n* = 17) Mean Change (95%CI)	Group Difference Mean Change (95%CI)	*p*-Value
**Body function** **and structure**				
UE strength (Rt)	1.2 (−1.8:4.0)	0.3 (−2.7:3.3)	0.8 (−3.7:5.3)	0.716
UE strength (Lt)	2.4 (−0.6:5.4)	0.4 (−2.7:3.5)	2.0 (−2.7:6.6)	0.389
LE strength (Rt)	6.0 (2.3:9.8) †	3.0 (−0.9:6.8)	3.1 (−2.8:8.9)	0.295
LE strength (Lt)	6.6 (3.1:10.1) †	0.7 (−2.9:4.3)	5.9 (0.5:11.4)	0.033 *
CSA-RF(Rt)	0.4 (0.2:0.6) †	0.03 (−0.2:0.3)	0.4 (0.01:0.7)	0.044 *
CSA-RF(Lt)	0.6 (0.4:0.9) †	0.2 (−0.1:0.4)	0.4 (0.04:0.8)	0.033 *
**Activity level**				
6MWT	30.3 (17.2:43.4) †	20.2 (6.7:33.8) †	10.1 (−10.4:30.6)	0.323
Sit-to-stand	3.5 (1.6:5.4) †	3.3 (1.3:5.3) †	0.2 (−2.8:3.2)	0.903
Borg scale	−0.2 (−0.7:0.4)	−0.1 (−0.6:0.5)	−0.1 (−0.9:0.7)	0.809
**Participation**				
Physical function	10.1 (4.0:16.2) †	5.8 (−0.5:12.1)	4.2 (−5.3:13.8)	0.371
Mental health	10.9 (5.1:16.8) †	1.0 (−5.0:7.1)	9.9 (0.8:19.0)	0.035 *

* Significant between-group difference (*p* < 0.05). † Significant within-group difference (*p* < 0.05). Covariates: age, UE strength (Rt), and CSA-RF (Rt and Lt). EG, experimental group; CG, control group; UE, upper extremity; LE, lower extremity; CSA-RF, cross-sectional area of the rectus femoris.

## Abbreviations

RA	Rheumatoid arthritis
CSA-RF	The cross-sectional area of the rectus femoris
6MWT	6 min walk test
SF-36	36-Item Short Form Health Survey
